# Getting to the bottom of research funding: Acknowledging the complexity of funding dynamics

**DOI:** 10.1371/journal.pone.0251488

**Published:** 2021-05-12

**Authors:** Kaare Aagaard, Philippe Mongeon, Irene Ramos-Vielba, Duncan Andrew Thomas

**Affiliations:** 1 Department of Political Science, Danish Centre for Studies in Research and Research Policy, Aarhus University, Aarhus, Denmark; 2 Faculty of Management, School of Information Management, Dalhousie University, Halifax, Canada; Indiana University Bloomington, UNITED STATES

## Abstract

Research funding is an important factor for public science. Funding may affect which research topics get addressed, and what research outputs are produced. However, funding has often been studied simplistically, using top-down or system-led perspectives. Such approaches often restrict analysis to confined national funding landscapes or single funding organizations and instruments in isolation. This overlooks interlinkages, broader funding researchers might access, and trends of growing funding complexity. This paper instead frames a ‘bottom-up’ approach that analytically distinguishes between increasing levels of aggregation of funding instrument co-use. Funding of research outputs is selected as one way to test this approach, with levels traced via funding acknowledgements (FAs) in papers published 2009–18 by researchers affiliated to Denmark, the Netherlands or Norway, in two test research fields (Food Science, Renewable Energy Research). Three funding aggregation levels are delineated: at the bottom, ‘funding configurations’ of funding instruments co-used by individual researchers (from single-authored papers with two or more FAs); a middle, ‘funding amalgamations’ level, of instruments co-used by collaborating researchers (from multi-authored papers with two or more FAs); and a ‘co-funding network’ of instruments co-used across all researchers active in a research field (all papers with two or more FAs). All three levels are found to include heterogenous funding co-use from inside and outside the test countries. There is also co-funding variety in terms of instrument ‘type’ (public, private, university or non-profit) and ‘origin’ (domestic, foreign or supranational). Limitations of the approach are noted, as well as its applicability for future analyses not using paper FAs to address finer details of research funding dynamics.

## Introduction

Ways and means to allocate research funding are considered one of the most influential elements in any attempt to govern contemporary public science. Funding is assumed likely to affect which topics get addressed, and the scope, content, direction, outputs and even potential impacts of public research [e.g. 1–4]. Funding secures the livelihood of researchers and is an indispensable prerequisite for almost all research [5]. Yet knowledge of how funding affects research remains fragmented and inconclusive. This stems in part from how research funding has been studied. In this paper we develop and test an approach that attempts to broaden how such studies are framed, by focusing on how researchers co-use funding at multiple different levels of aggregation.

Limitations of existing research funding studies mainly stem from insufficient attention to recent dynamics, i.e. to growing heterogeneity, complexity and related dynamic trends in contemporary research funding [5, 6]. There have been few attempts to acknowledge and identify the wide variety of potentially interlinked sets of funders now operating in scientific fields. Similarly, there has been insufficient exploration of the characteristics of the assorted funding instruments researchers may co-use to do research. Instead, most literature has typically studied single funding instruments and single funding organizations [see e.g. 2, 7] or has analysed strictly-confined national funding landscapes from a top-down/system-led perspective [4, 8–10]. However, the underlying assumptions justifying these approaches may no longer be adequate. They may even prevent deeper understanding of how researchers’ research funding can be composed and co-used, and how it influences research [11].

Existing approaches are particularly challenged by increasing developments towards more complicated and dynamic, border-crossing funding landscapes, where regional, national and supranational funders have proliferated from public, private and non-profit sectors [3, 5, 11, 12]. Despite recent calls for studies of this emerging research funding reality [5, 13] most scholarly claims remain based on general observations rather than on systematic, empirical studies [14]. An accurate understanding of how funding works, however, is a precondition for more realistic study of potential effects upon research of different kinds and combinations of funding. Improved understanding might also benefit research governance, revealing perhaps unsuspected funding synergies for policy action or discouraging use of possibly problematic research funding designs.

Research funding can also embody signals about needs that government(s), their agencies, industries and societies expect funded research to address [15]. An indicative example is research funded to address Grand Societal Challenges (SCs). For instance, in Europe, dedicated SC-related funding is expected to support research aiming to address pressing global problems (e.g. food security, energy security, public health, impacts of a changing climate). Policy or funder attempts to (re)direct such research to address these challenges is of course mediated by what research funding researchers actually mobilise to do their research. This may now include co-using more than one funding source, potentially leading researchers to face multiple, even conflicting signals from multiple funders.

The funding individual researchers actually encounter and (co-)use therefore is a crucial element. This is especially so because researchers are also an acknowledged obligatory passage point between research funding and research practice [16]. Approaches to study research funding, therefore, must explore the roles of funding from their viewpoint, rather than only from a coarse, system viewpoint. For this reason, our approach takes a researcher-led perspective. This leads to a ‘bottom-up approach’, entering the science system at the grassroots level of individual researchers and their funding, followed by increasing levels of aggregation of co-use of funding of collaborating researchers, and of a field of active researchers. In this paper, this approach is tested for two SC-related fields where multiple funders are known to operate, and are likely to provide varied funding instruments: Food Science and Renewable Energy Research.

For this approach, there are however different points in the research process through which funding could be studied. Funding could be studied as a research process *input*, as enabling certain research *practices*, or as associated with particular research *outputs*. To study funding as an input, for instance, document analysis of funding instrument characteristics could be attempted. To study funding of research practices, researchers could be surveyed or interviewed about their funding uses. Funding of varied forms of research outputs can instead be analysed. To demonstrate the broad applicability of a bottom-up approach, ideally several of these points would be studied together. For the approach testing purposes of this paper, however, we select only to study funding aggregation levels through research outputs in one of these forms: research publications. This selection is advantageous because funding of research outputs is reported in a standardized fashion. Studying non-paper formats of research output could run into difficulties, e.g. variation in funding reporting protocols. By contrast, in publications, for over a decade researchers’ have routinely self-reported certain funding details, as funding acknowledgements (FAs). To provide a manageable scale for the test, FAs in papers are selected only for researchers affiliated to research organizations (ROs) in three countries (i.e. Denmark, Norway, the Netherlands). This test selection gives both sufficient variety, as these are small, similar yet still intensive research funding contexts, and manageable scale.

Overall this paper aims, first, to frame a bottom-up, funding of researchers-led perspective on research funding dynamics. Second, it will empirically test an analysis of research funding using this perspective through the specific example of FAs in researchers’ publications. The approach is based upon three analytical levels:

- ‘Funding configurations’ of funding instruments co-used at the level of individual researchers.
- ‘Funding amalgamations’ of funding instruments co-used at the level of collaborating researchers.
- ‘Co-funding networks’ of funding instruments co-used at the level of all researchers active in the field.

The paper is structured as follows: the next section justifies why adding a bottom-up perspective is needed and could improve understanding of research funding dynamics. This is followed by a section presenting the bottom-up approach. The strengths and weaknesses of FAs as a data source to illustrate the approach are then reviewed, and the added value of using FAs for a multi-level approach to study research funding dynamics is described. The subsequent section describes the test case selection, research field delineations, FA data collection, cleaning and coding. Findings are then presented for funding configurations, funding amalgamations and field co-funding networks based on the FA data. Finally, we reflect on how the bottom-up, researcher-led approach to funding co-use contributes to understanding research funding dynamics, and conclude with implications for science policy and further research directions.

### Why add a bottom-up perspective on research funding?

To understand the value of adding a bottom-up perspective, it should be noted that most previous approaches to research funding studies have relied on a top-down or system-led perspective. This means that to observe funding, studies have focused on features of funding aggregated for national or otherwise geographically confined funding landscapes [e.g. 8, 9, 17, 18]. Alternatively, they have examined effects of single funding organizations or single funding instruments within the science system, and attempted to isolate effects for just one funding source [e.g. 10].

There are sound, pragmatic, methodological and conceptual reasons for this situation. Clear research field delineations and geographical boundaries for funders’ assumed spheres of operation and influence, drawn from the top-down, can isolate effects of specific funding organizations or instruments from many other extraneous, mediating factors. They also likely service the interests of funders aiming to document and evaluate effects of their own specific investments. Likewise, a national focus may suit vested interests of national policy makers and stakeholders [11] in legitimising particular uses of public resources. Such analyses may be valid for specific research objectives, e.g. to delineate the portfolio of a single funding organization. However, additional, complimentary approaches are needed to generate broader understandings of researcher-level funding dynamics.

Traditional positions have also generated important insights. Top-down perspectives–typically based on data from OECD Main Science and Technology Indicators (MSTI), Eurostat, national statistics or large cross-country studies–afford broad overviews of central components of national funding systems. These enable comparative insights into important characteristics at country level, such as volume of R&D funding, balances between institutional funding and project funding, distribution of funding between disciplines, and allocation mechanisms for institutional funding [e.g. 4, 9, 19]. Studies of individual funders and single funding instruments have also provided insight into key characteristics and mechanisms, like how selection procedures for funding instruments might enable support of exceptional research [e.g. 2, 20] or how funding properties, and properties of the research funded, become interdependent for some funding instrument classes [e.g. 7, 21, 22].

However, such studies also build on two key assumptions that may have become increasingly questionable. First, they assume national funding landscapes mapped and studied from the top down provide a relatively *complete* picture of funding actually mobilised by researchers working within a given national system. Second, they assume individual funders and funding sources mainly operate *in isolation*–and hence their operations and effects for researchers using their funding can and should be studied in a kind of vacuum. These assumptions may once have been appropriate, but they are now challenged by multiple, interrelated developments in science and science policy.

First, funding has shifted from internal/institutional to external/project-based in most countries [2, 23–26]. Second, the scope of research policy has broadened to direct funding towards more diverse goals, e.g. promoting ‘excellence’ in science, providing solutions to economic and social problems, and fostering technology and innovation [6]. Project funding instruments and funders have thus multiplied and become increasingly differentiated [9]. In addition, public knowledge production has become more integrated into society, creating an ‘extended peer community’ [27, 28] where non-governmental actors are involved as funders, collaborators or stakeholders. Third, increased globalisation of science has made international research collaboration far more widespread [11], increasing the importance of non-national funding. This includes different types of EU allocations, funding from public, non-profit and private sources, and funding from other countries either directly or indirectly (i.e. through collaboration with international colleagues). All three of these challenges are potentially in play simultaneously. Consequently, the organization of science increasingly ceases to follow disciplinary and/or national borders, and instead features trans-national, trans-sectoral, multi/inter/trans-disciplinary academic communities or fields, and is characterised by both pervasive geographic and research field boundary-crossing [11, 29].

Contemporary research at individual, collaborative and field levels, therefore, also is likely to rely on multiple, heterogeneous funding, and funding co-use. Blends of funding instruments mobilised from the bottom up are a part of this development of increasingly multi-level, multi-actor governance systems, and will differ substantially from traditional perceptions of research funding driven by national, public authorities [3, 5, 6]. For these reasons, studying funding dynamics must now consider not only how national funding systems are designed from the top down, but also how funding is mobilised and even co-used by researchers from the bottom up.

While these challenges are increasingly acknowledged, the majority of literature has largely not addressed them. However, some recent studies have begun to call for a ‘reality check’ [see e.g. 5, 12, 13, 30]. It is becoming recognised that these research funding dynamics require new approaches, and the limitations of traditional approaches are increasingly highlighted [6, 12, 13]. It is argued that research funding might be better treated as sets of interlinked geographical and/or research field spaces of interaction between different layers of research funders and performers [12]. These spaces are being recognised as not exogenously defined by organizational or geographical distinctions. Instead they need to be empirically observed [12]. Additionally, the increasing importance of charities, supranational funding agencies [20, 31] and the emergence of new funding schemes for science [32] all need to be explicitly considered more. New approaches tailored to capture these more complex funding dynamics are, therefore, seen to be necessary [5].

Most previous studies are further challenged by the analytical level that is adopted. The typical emphasis on aggregating at national level may mask important field differences even within countries. These can include, e.g. large differences across scientific fields in relation to the balance between institutional and project funding [18]. Similarly, the roles of non-public or non-national funders may vary across fields taken within the same national system. In some fields, public sources may dominate whereas private foundations, patient organizations or companies may in others. Field-specific funding organizations or funding instruments may be marginal for a national picture but nevertheless could play substantial roles at lower levels of aggregation. Similarly, the importance of non-national funding may vary across fields.

National or international research-related statistical organizations are also seldom able to capture the full extent of research fields, to provide usable field delineations for funding studies. Empirically-delineated research fields often will not correspond to the organizational or disciplinary categories used by nation-oriented bodies. Even statistical data at disaggregated levels, if originally framed by national levels, may introduce demarcation problems. Therefore, as science organization and funding have become more complex over time, unsurprisingly the availability of appropriate research funding statistics has struggled to keep pace [6].

Overall, how science and policy have developed has increasingly challenged traditional top-down, system-led perspectives on research funding. Coupled with field delineation and statistics shortcomings, this generates numerous, problematic assumptions about research funding. The available research funding literature has seemingly not yet fully embraced the implications of these changes. This justifies exploration of a new perspective and approach better suited to tackle these current funding dynamics, and to complement the strengths more traditional approaches can still provide (e.g. regularity, standardization).

### A bottom-up approach to research funding dynamics

To begin, the bottom-up approach to research funding dynamics considers funding of the smallest knowledge producer in any research field, i.e. individual researchers, and their research funding instruments. A ‘funding instrument’ is understood as the lowest identifiable level, discrete resource unit provided by any funder (e.g. a specific grant from an internal or external funder). Individual researchers work at a research organization and, considered within a specific window of time, may sometimes co-use a set of funding instruments, i.e. a ‘funding configuration’. An individual researcher’s configuration could fund, e.g. writing a paper, or building equipment, conducting fieldwork, disseminating research results and so on. The next, middle level of aggregation considers funding of knowledge production where researchers collaborate, i.e. funding instruments co-used by collaborating researchers. Co-use may not mean researchers use each other’s funding here, simply that funding has somehow been used to undertake a collaborative research activity. This could include, but is not limited to, co-authoring a research publication. This is a ‘funding amalgamation’. The top level considers funding of knowledge production at level of a research field. This aggregates co-used funding instruments identified in the previous levels, considering now all the researchers active in a research field. This delineates the ‘co-funding network’ of that research field. Scientometrics-based study of co-funding typically only presents this level, and does not frame configurations or amalgamations to study co-use funding by individual or collaborating researchers.

Table 1 contrasts the shift in perspective these three levels of the bottom-up approach provide, relative to the traditional, top-down approach to research funding. Separately, it should also be noted this approach also differs from existing ‘bottom-up’ approaches in scientometrics used to assess research performance [see e.g. 33].

**Table 1:** Contrasting top-down and bottom-up approaches to research funding.

	Top-down	Bottom-up
**Perspective**	System-led, nationally-bounded, policy/funder framing of field	Researcher-led, geographically unbounded, researcher activity-based delineation of field
**Analysis of funding dynamics**	National funding landscapes	Co-funding networks
	Single funding organizations	Funding amalgamations
	Single funding instruments	Funding configurations

**Fig 1:**
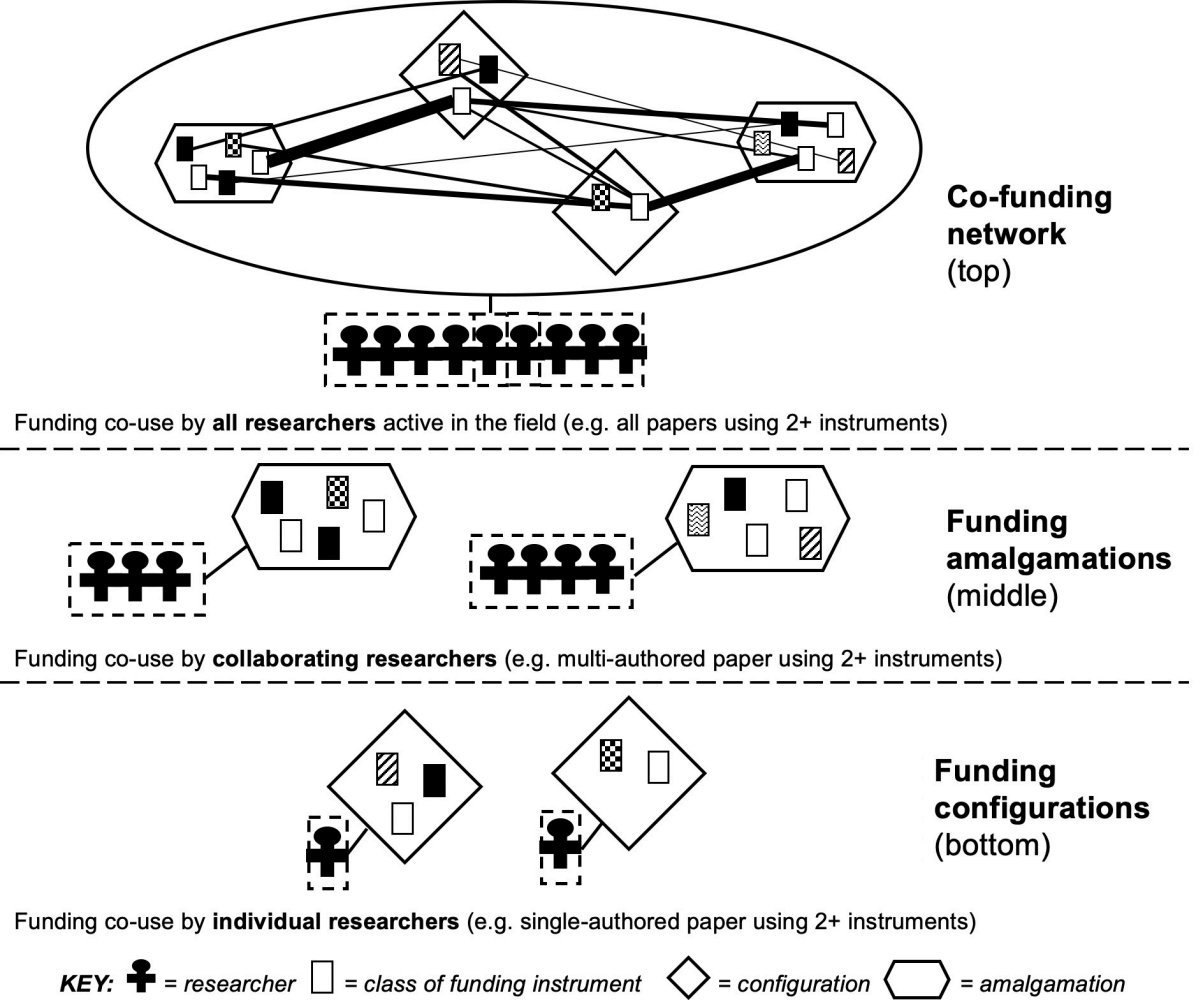
Three levels of a bottom-up approach to research funding dynamics. Line thickness between instruments (boxes) of different classes (e.g. type, origin, providing funder) in the co-funding network represents occurrences of instrument classes that have been co-used.

Fig 1 visualises the bottom-up approach’s three levels–i.e. funding configurations, funding amalgamations, and field co-funding networks. The bottom, ‘configurations’ level registers the distinct sets of funding instruments co-used by individual researchers; the middle, ‘amalgamations’ level registers sets of multiple funding instruments co-used, in some way, by collaborating researchers; and the top level is the ‘co-funding network’, registering all funding instrument co-use instances by all researchers active in the field (i.e. all instances when two or more instruments have been co-used to support individual or collaborative research activity, thus aggregating all forms of configurations, and all forms of amalgamations, within the given research field). The network nodes are instruments of different classes, that can be characterised using appropriate codes (e.g. public, private, national, supranational). The thickness of ties between instruments counts the occurrences of those specific linked instrument classes across the field. This is then based on all instances of instrument co-use by all researchers active in the given field (i.e. all configurations of two or more instruments, and all amalgamations of two or more instruments). Particular instrument classes may then be observed as co-used together more than others (e.g. public instrument-public instrument co-use may be the most common form in some fields; public-private-not-profit, in other fields) or instruments can be grouped by the funder that provided them, to study that aspect. The approach overall captures three different levels of funding instrument co-use. It enables study of how different kinds of funding are blended, in various ways, by individual researchers, by collaborating researchers, and across an entire field of active researchers. (To reiterate, this approach does not study isolated uses of single funding instruments by researchers–i.e. *not* involving co-use. This would be a separate design, and is not undertaken in our multi-level approach, which focuses exclusively on co-use.)

### Funding acknowledgements to study funding co-use dynamics

To empirically test our bottom-up approach in the study of funding of researchers at different levels of aggregation, we look at funding of research outputs based on funding acknowledgements (FAs) in publications. We review previous uses of FAs in the literature, and emphasise that the approach developed in this study uses FAs to validate its multi-level framing of funding, and not to study research performance, e.g. from a scientometrics perspective, or to advocate that these funding levels can only be studied via publication FAs. Functional and behavioural-related limitations to using FAs as a data source to test our approach are noted. This is followed by a description of the added value of using FAs to study funding co-use dynamics from the bottom-up.

#### Previous FA-based studies

Numerous FA-related studies have been produced since FA data became systematically available in large-scale publication databases from late-2008. Some of these have highlighted co-use dynamics. FAs have previously been used as a data source to demonstrate co-funding patterns [e.g. in nanotechnology; see 34, 35]. Analysis of FAs has also shown multiple funding instruments can be co-acknowledged in the same paper, e.g. a publication can acknowledge support from mixes of both public and private funders [36]. FAs have also been used to map citation scores of research outputs affiliated to particular research organizations within a geographical boundary, e.g. the continent of Africa, to rank relative performance of individual research funders and/or performers [37].

Even when such studies attempt to delineate apparent ‘funding landscapes’ derived from FAs however, they strictly speaking constitute research performance landscapes, i.e. mapped primarily to assess research performance, and to address which individual research organizations, countries or funders perform or fund the highest cited research. Alternatively, FAs have been used to delineate which research themes and topics are funded across a particular field or geographical area [38] to inform research prioritisation and policy. In other words, such research typically uses FAs to assess performance or priorities–i.e. for evaluative or descriptive purposes [33], and does not typically disaggregate multiple levels of funding co-use, which is our approach.

#### Limitations of using FAs as a data source

Use of FAs in previous literature has also revealed some limitations to their use as a data source. Reviewing these studies, a first class of limitations can be seen to be functional in nature (Table 2).

**Table 2:** Functional limitations of FAs as a data source. [See 34–3,6, 39–50].

Issue	Explanation
Availability	Not always present because some publishers and funders do not mandate them.
Correctness	Self-reported by authors with possibly imperfect recall, which can be compounded by weak oversight or accountability regarding FA accuracy and consistency. Possibilities arise for potential indexing errors in publication databases.
Standardisation	Neither uniformly structured nor all-encompassing, because different publishing outlets apply differing acknowledgement practices and templates or even provide no guidance about FAs at all.
Quality	Often require manual cleaning and disambiguation of misspelled and/or inconsistently abbreviated or translated funder names, and may have similar errors for grant numbers. Funder names also change, new funders enter and old ones exit over time.
Completeness	Information about funding amount(s) and specific grant numbers is often missing.
Usage	Offer limited reliability to link funding with performance/impact of publications, e.g. via citations, or to attribute specific outputs to particular grants, because published findings may follow after funding or vice versa, and most grants are usable in highly flexible ways (e.g. in what research and publications are pursued versus what was promised).
Scope	Largely available only for papers published after August 2008, when Web of Science (WoS) began to index FAs, then with more consistency from 2009 (Scopus began to index in July 2013). FAs indexed in databases still often have limited coverage of non-English paratexts.

A second class of limitations to using FAs as a data source can be considered as caused by researchers’ acknowledging behaviours. They exist because although funders and authors increasingly consider providing FAs in papers to be mandatory or at least good practice, compliance can vary from researcher to researcher, across researcher country and language groups, or across fields. Consequently, FAs can under- or over represent funding data. FAs may also not reveal certain kinds of funding information, or can equate all funding so it seems to be of equal importance for the published research, when in fact it is not (see Table 3).

**Table 3:** Limitations of FAs as a data source due to author acknowledging behaviours. [See 35, 36, 39, 40, 43, 44, 47–4,9, 51–53].

Issue	Explanation
Under-representation	Funding may be under-represented, as not all sources are always self-reported. Funders may be unacknowledged if authors win multiple sources to research very similar topics but do not wish this to be known. Commercial and charitable sources may be undisclosed due to vested interests or secrecy. Use of institutional/internal university funding is commonly underreported.
Over-representation	Funding may be over-represented, e.g. to boost apparent outcomes of grants and/or author reputations (by over-emphasizing or even spuriously naming prestigious funders) including when little or no relationship exists between acknowledged funding and the actual published research.
Obscuration	Difficult to disambiguate to robustly attribute funding to authors in multi-authored papers that acknowledge many funders, unless authors make this information explicit (i.e. estimations are thus often used).
Equation	FAs shed little light on the relative importance of sources acknowledged, because authors often omit such information. All acknowledged funding may therefore superficially appear to have equal significance or weighting, when in reality funding has varied in terms of size, type, origin or other important characteristics.

#### Strengths of using FAs as a data source

Despite these limitations, using FAs for a papers-based test of the bottom-up approach usefully enables delineation of funding configurations (funding of outputs produced by individual researchers), funding amalgamations (funding of outputs produced by collaborating researchers) and co-funding networks (funding of outputs by all researchers active in a field). These would otherwise be difficult to map without using FAs [40, 54]. Other methods could have challenges regarding resource intensity, intrusiveness and coverage. For example, instead of using publication FAs, researchers in a field could be surveyed and asked to self-report their funding instruments or configurations. Research groups, teams or networks could be surveyed about funding amalgamations. Such surveys, however, could have issues of memory recall and data completeness, and could be resource-intensive.

Selecting a FA-based method primarily to test the bottom-up approach instead has clear advantages. First, for the test, compared to other methods, e.g. surveys or interviews, FAs are pre-existing paratexts in papers and can be automatically extracted, at scale, from publication databases. This means using FAs is cost-effective. It also enables large-scale, unobtrusive study of funding across all three levels of the bottom-up approach. Second, FAs are funding data self-reported by researchers themselves, rather than via third-party sources, such as funder databases or other repositories. These may not be as direct or comprehensive as FAs. Third, a FA-based approach is scalable and repeatable longitudinally. For studies beyond our current test, they could be used to sample multiple time periods with little marginal cost, if relevant periods are covered by publication datasets. Fourth, as publishers and publication datasets adapt to and advocate their increasing use, FAs may become more available, consistent and stable. As a data source they may become more significant for using a bottom-up approach over time. The primary drawback, however, is that using FAs artificially restricts analysis of funding co-use dynamics to study of funded papers. It excludes studying other co-use instances, which would need separate research, exploring beyond just publication-related research activity.

#### Using FAs for a bottom-up approach to research funding

To operationalize use of FAs to test study of research funding dynamics from the bottom up, the starting assumption is that individual researchers can co-use multiple funding instruments, which they acknowledge via FAs, even in single-authored research (i.e. these would then be funding configurations). Funding instruments can become more aggregated within collaborative research activities. These mix–to an unknown greater or lesser extent–some or all the different funding instruments brought in by every researcher (i.e. funding amalgamations). These are evidenced via FAs in multi-authored publications, where co-use includes researchers using separate funding instruments to co-author the paper and/or researchers’ sharing funding instruments. Finally, co-use of funding instruments across a field of researchers becomes highly aggregated. This can be delineated from FA data as a field co-funding network. To produce each level, associated analytical steps are required. These are careful research field delineation, name disambiguation, cleaning of all acknowledged funding instruments, and analytically-informed coding of FAs to classify funding instruments.

To conceptualise this coding, the bottom-up operationalization approaches FAs as traces of complex, adaptive, global contemporary science, potentially involving many funding actors. This acknowledges that paper FAs as a data source report actual funding (co-)used by researchers in a way that is indifferent to boundary/border issues. That is, they naturally accommodate the reality that researchers can and do reach both below and above national levels to obtain funding instruments, and to ‘configure’ or ‘amalgamate’ them together in ways that can transcend geographically-constrained, topic or sector-specific funding patterns [11]. Nevertheless, an ability to study any national system-related patterns can still be retained. This is done by registering the author affiliation(s) of researchers. For this test, this will be to one or more of the three selected countries (Denmark, Netherlands, Norway), taken from reported author affiliation(s) in publications, in the two test research fields (Food Science, Renewable Energy Research).

There are further issues to note for the operationalization. These concern attribution. For funding configurations, FAs in single-authored publications do provide directly attributable data on funding instrument co-use by individual researchers. In a paper with only one author, if multiple instruments are reported, they unequivocally have been co-used. However, funding amalgamations are operationalized via funding of multi-authored publications. FAs usually provide an undifferentiated data string, without funding-author attribution. They may not attribute each instrument (FA) to an author. It cannot be robustly determined if all the acknowledged funding belongs to only one author (e.g. other authors acknowledge or have no funding). Funding amalgamations can therefore vary. They may involve fusion, where all authors producing the paper are supported by one or more shared funding instrument. They may feature juxtaposition, where authors do not mix funding, but use their own, separate instruments. The amalgamation does not aim to describe how acknowledged funding instruments have been mixed; this needs exploration via other methods. It registers only an analytical level of funding co-use–broadly understood–as funding contributing, in some as yet unknown way, to the act of collaborating to produce a paper.

Publication FAs also typically provide only funder and/or funding instrument names. Therefore, the operationalization needs to code funding instruments. This requires selecting instrument characteristics that are analytically meaningful to label. Based on previous literature, ‘type’ and ‘origin’ were selected to be coded for our test (although others could be developed in future). Type distinguishes whether funding instruments are from a public, private, non-profit or university funder. This can classify the often distinctive kinds of funding instruments provided by these bodies [see 5, 10, 55–57]. Deliberately, both internal and external funding ‘types’ are coded. The role of university/internal funding (e.g. institutional/block grant funding of salaried research time, specific internally funded research activities) is quite underexplored [see 30], so should be included

Coding origin follows established practices for FAs [e.g. 36]. It distinguishes domestic from foreign or supranational funding instruments. This enables specific characteristics to be flagged, e.g. domestic (i.e. national or sub-national) funding instruments can differ from foreign funding (non-domestic national or sub-national) and from supranational funding in analytically relevant ways. This may be due to these funders’ geographical scope of operation or focus on particular challenges, economic and/or societal missions (e.g. contrasting the EU, multinational corporations, and international non-governmental organizations).

### Selecting the test cases

Our two test cases, the Renewable Energy Research and Food Science research fields, were selected to test the approach. This is because they have varied funding and assorted funders to delineate, due to these fields having a wide breadth of research topic/keyword coverage [e.g. see 58]. They also feature both fundamental science and applied research, and long traditions of involving industry, public authorities, research users and other collaborators. The two fields are also thematically oriented. This means they can engage grand SC themes, e.g. United Nations Sustainable Development Goals (SDGs) and related policies and politics. These test fields do not represent generally the global science system. They are however, appropriate, dynamic and multi-faceted candidates to test the bottom-up approach. They represent a confirmatory case selection [59]. The two fields provide similar heterogeneity, yet sufficient variation to enable testing of the approach.

Researcher author affiliation(s) were selected to be research organizations (ROs) in Denmark, the Netherlands and/or Norway. This retains an ability to explore the role of national contexts, even when adopting a bottom-up, country-crossing perspective. These three selected countries are all small, advanced, Western European economies. They have similar Humboldt-inspired university systems, relatively high shares of institutional funding, and traditional public research councils [17]. At the same time, they have country specific funder/funding variations. E.g. Denmark has relatively clear demarcation between scholarly and societally oriented funders; Norway is more centralized with a broad, unified research council. All three affiliation countries also have active Food Science and Renewable Energy Research sectors.

#### Field delineations and time horizon selection

To delineate the two test research fields, Whitley’s notion was adopted of ‘intellectual fields’ as a ‘broader and more general social unit of knowledge production and co-ordination’ than disciplines [60]. Research ‘fields’ were considered as fairly broad units of knowledge production. They can engage activities outside academia, knowledge co-production with industry or other societal collaborators, and still involve public research organization-related social and organizational structures.

To develop the data collection process, all Web of Science (WoS) publications were retrieved with at least one author with any affiliation to a research organization located in Denmark, the Netherlands or Norway. Renewable Energy Research and Food Science publication datasets were created using a core/expansion approach. The core datasets for the two fields included all papers meeting all three criteria of: containing at least one of our search terms; being published in a relevant journal; and being part of a relevant article-level cluster.

The list of search terms used for each field can be found in S1 File. The relevant journals from each field were selected by going through the list of journals included in relevant WoS subject categories (i.e. “agriculture, dairy & animal science”, “agricultural economics & policy”, “agriculture, multidisciplinary”, “agricultural experiment station reports”, “food science & technology” and “agricultural engineering” for Food Science; and “energy & fuels” and “green & sustainable science & technology” for Renewable Energy Research; previous energy research [58] was also consulted) or by containing one of our search terms in their title. In the Centre for Science and Technology Studies (CWTS, Leiden, Netherlands) database used for this paper (that included high resolution funding data and author-name disambiguation [61]) articles were clustered based on citation relationships [c.f. 62, 63]. Clusters with at least five publications containing one of our keywords were screened to identify those clearly related to the fields. The relevant journals and article-level clusters are also included in S1 File.

These two core datasets were then expanded by including all papers meeting any three of the following five criteria:

- contained at least one of the search terms;
- published in a relevant journal;
- part of a relevant article-level cluster;
- cited or is cited by a publication in the core set;
- shared an author with a publication in the core set.

Finally, in each field dataset, all papers in the micro-clusters for which more than one third of publications had already been included at that point were also included.

To include publications and their FA data for a reasonable period, the dataset time horizon was selected to be 10 years, i.e. all field publications 2009–18. This had the advantage of starting the datasets soon after WoS began to systematize and make available suitable FA metadata. No specific time criterion was used for author inclusion, i.e. for whether a particular author-researcher had to appear multiple times within the period to be considered ‘active’ in a field. This process produced a publications dataset, covering all six field/affiliation cases, which could be separately studied by filtering fields and/or countries. Gephi software was used for network visualizations.

#### Disambiguating and coding funding instruments from FA data

All FA data in this dataset of publications was cleaned and coded. FAs were taken from the ‘funding agencies’ metadata field of WoS records. This provided funder organization names, names of sub-divisions of these funders, and sometimes funding grant numbers or funded project names (i.e. specific details of funding instruments).

Initially, 25,522 distinct funder strings were automatically retrieved from the FAs. Funder strings that occurred at least twice in the dataset were cleaned and disambiguated. Sub-divisions of a funder found in that set were grouped under the main named funder (e.g. diverse funding instruments of the European Commission were grouped). This process merged 19,140 (75%) of the 25,522 original funder strings into a cleaned list of 2,363 unique funding organizations to analyse. Throughout, data errors encountered during the extraction process were addressed, e.g. cleaning funder name variants.

Given the limitations of FA data we have already noted, funders had to be disambiguated and classified so as to code their associated, acknowledged funding instruments. This was done using the selected data labels of type and origin. Four values were used to code type of instruments:

- Public–typically instruments from research councils and public/state authorities like ministries and agencies (but including supranational organizations like the EU, and regional and local authorities).
- Private–predominantly instruments from companies/corporations.
- Non-Profit–instruments from private non-profit organizations, patient organizations, national and supranational non-governmental organizations (NGOs) and related others.
- University–internal funding directly from a university (most likely either institutional funding or competitively awarded internal university funds).

Three values were coded for origin:

- Domestic–instruments originating from Denmark, Norway or Netherlands funders (respective to author affiliation).
- Foreign–instruments originating from all other countries, except the domestic country.
- Supranational–instruments from funders like the EU, OECD, UN agencies.

Table 4 shows the final total number of articles collected in the full dataset, the number and percentage of papers with at least one acknowledged funder (i.e. instrument detail), and the share of FAs able to be coded for type and/or origin (i.e. between 67.9% and 77.8% for type; between 70.7% and 81.2% for origin). There was an average of 2.3 FAs per paper, for the full dataset of papers with FAs (i.e. 55,089 FAs across the ~23,000 papers with FAs). Non-coded FAs were sampled to check for systematic bias, and their characteristics were found to be acceptably similar to the coded proportion.

**Table 4:** Coded articles and FAs for Renewable Energy Research and Food Science, 2009–18.

	Publications				Funding acknowledgements (FAs)		
Case	Number of authors	% with FA	N^o^ of articles	% with FA	N^o^ of FAs	% identified type	% identified origin
Energy-A-DK	14,497	83.5	6,960	73.7	11,817	77.8	81.2
Energy-A-NL	18,136	81.2	7,390	70.0	12,417	71.2	74.4
Energy-A-NO	8,069	84.3	3,369	76.8	6,540	72.1	75.0
Food Science-A-DK	14,488	83.4	5,111	77.5	10,197	70.9	74.8
Food Science-A-NL	17,946	80.0	6,251	69.9	11,057	67.9	70.7
Food Science-A-NO	10,950	83.6	4,322	79.3	8,920	70.2	72.7
Full dataset	67,999	82.8	31,514	73.5	55,089	71.5	74.6

A = affiliated (these are not country cases); DK = Denmark; NL = Netherlands; NO = Norway.

**Fig 2:**
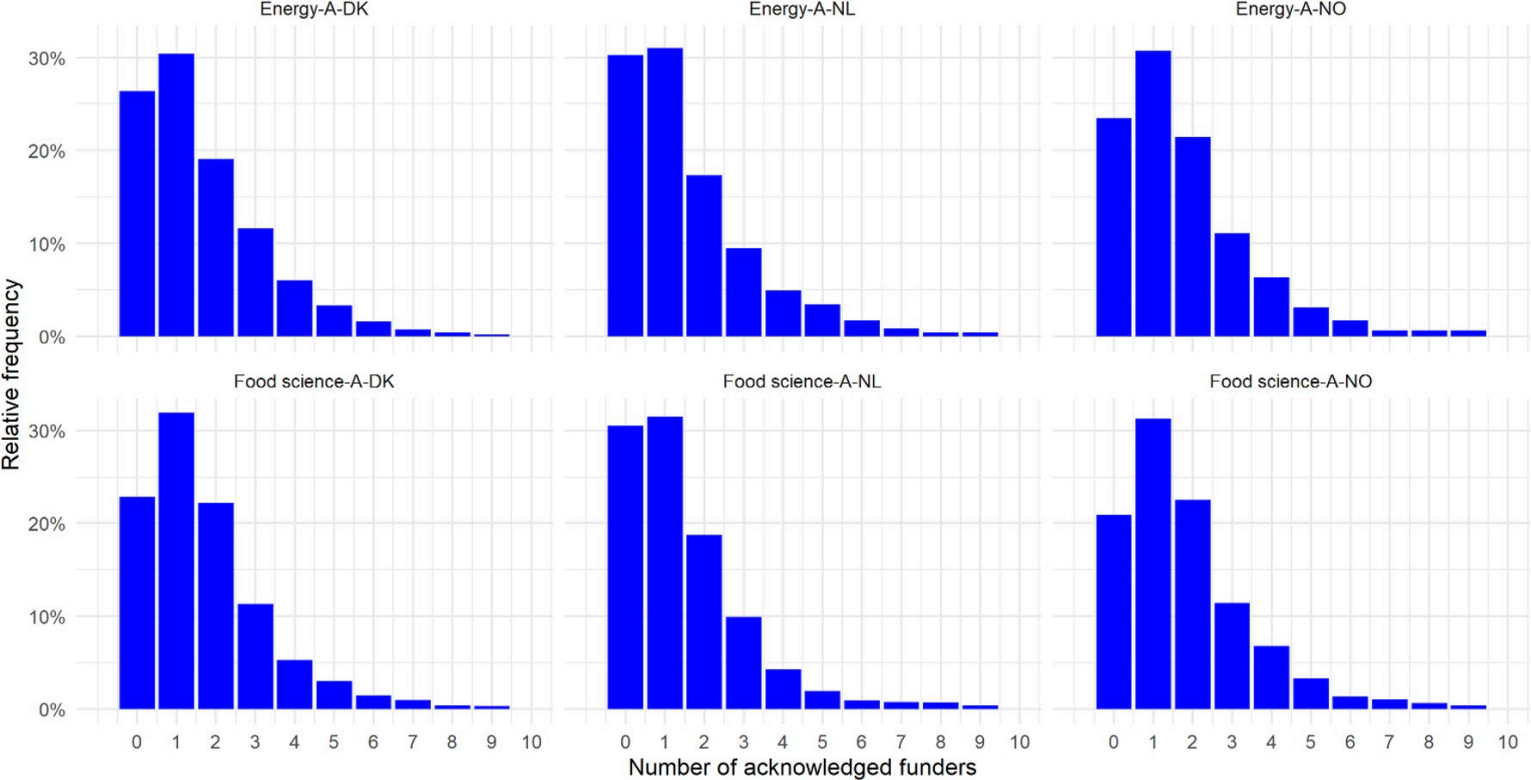
Frequency distributions of numbers of FAs across papers, 2009–18.

The sub-sample used to obtain configurations, amalgamations and co-funding networks was derived after exploring the number of acknowledged funders across all publications (see Fig 2). The aim was to exclude articles with no FAs or only one FA. Across the cases, this left 35–45% of the articles from the full dataset with two or more FAs (i.e. instruments). Of these papers, 0.7% were single-authored (funding configurations) and 99.3% were multi-authored (funding amalgamations). Co-funding networks were obtained by including both single and multi-authored papers with multiple FAs.

### Illustrative findings

Findings from testing the approach are now presented. For funding configurations, this presents patterns of co-used (i.e. co-acknowledged) funding instruments at individual researcher level from single-authored publications. For funding amalgamations, patterns of co-used funding instruments, number of FAs, and number of authors for collaborating researchers, are presented to demonstrate dynamics for multi-authored papers. Finally, field co-funding networks are presented for Renewable Energy Research and Food Science, respectively. This combines all possible affiliation countries and co-use funding patterns from both single and multi-authored papers. Country affiliation-specific networks are also presented, to demonstrate the approach can still illustrate nation-related features for study. Across all three levels, funding instrument type (public, private, non-profit, university) and origin (domestic, foreign, supranational) are explored. This presents our approach to co-use of funding by researchers, conceived of as three increasing levels of aggregation–funding co-use by individual researchers, co-use by collaborating researchers, and co-use by a field of researchers. This illustrates co-use of funding by researchers through the specific lens of papers as a research output. As stated earlier, funding co-use could be explored for different research activities, but still aggregated at these three levels. Throughout, it should also be stressed these are illustrative findings to test the approach, not a comprehensive overview of Renewable Energy Research or Food Science.

#### Funding configurations

The bottom level illustration is obtained from single-authored papers with two or more FAs, so presents individual researcher level, funding configurations. Table 5 shows funding configuration patterns. This is in terms of number of funding instruments being configured. From this observation, two findings become evident. First, funding configurations exist in the data. There is acknowledged co-use of funding instruments even in individual researchers’ lone publication outputs (FAs were checked to confirm that each FA did in fact refer to a distinct instrument). Second, in both fields, funding configuration scales vary–even for this small sub-sample. Most configurations featured two instruments (62 out of 90 papers). Others had three, four or even five or more instruments reportedly co-used for a single-authored publication.

**Table 5:** Funding configuration patterns in the two fields by number of FAs, 2009–18.

Field	N^o^ of authors acknowledging funding configurations (i.e. multiple FAs)	N^o^ of single-authored papers with 2 FAs	3 FAs	4 FAs	5 or more FAs
Energy	46	40	8	2	2
(A-DK, A-NL, A-NO)					
Food Science	29	22	12	3	0
(A-DK, A-NL, A-NO)					
Both	75	62	20	5	3

A = affiliated (these are not country cases); DK = Denmark; NL = Netherlands; NO = Norway.

This bottom level, for this illustrative test, is only a small sub-sample of the full dataset. It has only 75 authors and 62 single-authored publications, acknowledging two or more funding instruments. It should be stressed this does not demonstrate that some representative portion of researchers in these two fields have funding configurations. Instead, the data verify that it is *valid and important* to consider, and to focus upon dynamics of co-use of funding, at individual researcher level–i.e. it is valid to study this analytical level, when exploring uses and roles of funding for research.

A limitation of our FA-based test of the approach is that funding configurations are only observable for single-authored papers here. In highly collaborative fields, single-authored papers are likely rare, and multi-authored papers more common. Separate study could use the insight that funding is co-used at individual researcher level, to study individual researchers via a different research activity. This could, e.g. be individual researchers co-using funding for building equipment, for undertaking fieldwork, or to support policy engagement activity. Even with a small sub-sample, there is configuration variety, in terms of funding instrument type and origin. We argue then these findings warrant such additional explorations. Separate qualitative work on single-author researchers in this dataset has also highlighted variety in reasons for, and mobilizations of co-used funding instruments, see [64].

The main finding here is not that these particular configurations are representative of individual researcher co-use of funding in these specific fields. Rather it suggests there are potentially interesting dynamics at this level that warrant the inclusion of funding configurations in studies of funding moving forward. Most importantly, these dynamics go unexplored if this level of analysis is *excluded*. However, separate study of research activity–not limited to publications/FAs–would be needed to understand exactly how and why individual researchers co-use funding instruments, for various research purposes. Similarly, exploring which patterns of funding configurations are prevalent in any given field would require alternative methods. This is because FAs cannot study individual researcher co-use dynamics that happens anywhere other than in single-authored papers.

Within these limitations, and for our specific FA-based findings, we see configurations of ‘public’ instruments are the most common co-use by individual researchers, in terms of funding instrument ‘type’. However, there are also ‘public-private’ and ‘public-university’ configurations, and ‘non-profit’ instruments configured with all three other types by individual researchers. Similarly, there are configurations with mixed ‘origins’. The most common is ‘domestic-domestic’. This seems an interesting indication of even single-authored research needing to draw upon multiple domestic instruments (e.g. multiple national funders). However, there are also ‘domestic-foreign’, ‘domestic-supranational’ and ‘foreign-supranational’ configuration varieties.

Overall, the sub-sample shows funding configurations exist, and that they can vary in terms of type and origin. This is even when only exploring a small number of cases. Taken together, this suggests configurations are a valid analytical level to consider in studies of funding dynamics in a broader sense (and this would eventually be broader than only FA/publication-based data collection).

#### Funding amalgamations

The middle level in our bottom-up approach presents amalgamations of instruments co-acknowledged in multi-authored papers (with two or more authors, and two or more FAs). These funding amalgamations indicate that collaborating researchers co-use a wide range of different instruments. This is seemingly broader than how co-use occurs in funding configurations of individual researchers. As we have stated, it cannot be determined–through FAs alone–whether these amalgamations are fused or juxtaposed instrument co-use. However, these illustrative findings are promising to validate the approach, and an emphasis upon this level of funding aggregation. They suggest more funding variety can be seen in funding amalgamations than in funding configurations. This tentatively offers interesting insights by including this level of funding aggregation in studies. It also indicates potentially important funding dynamics may arise from co-use of instruments at the level of collaborating researchers (here addressed only for multiple authors co-authoring a paper).

Patterns of funding amalgamations are presented by type of instrument in Table 6. The most prevalent amalgamation type is different public funders being co-acknowledged. In Renewable Energy Research for Netherlands- and Denmark-affiliated publications, this ‘public-public’ funding amalgamation type accounted for around 60% of FA cases (60.9% and 58.8%, respectively); for Norway-affiliations, it represented 43.4%. For Food Science, across the same three affiliations, the pattern was different: 44.9% for Netherlands-affiliated, 44.2% for Denmark, and 50.4% for Norway. Such patterns might suggest certain co-influences of these types of funders for these collaborative research outputs, and that influences may vary by field.

**Table 6:** Funding amalgamation patterns by instrument type, 2009–18.

		Renewable Energy Research				Food Science			
		Non-profit	Private	Public	University	Non-profit	Private	Public	University
**A-Denmark**	Non-profit	1.1	1.5	8.9	1.2	2.5	4.8	17.5	4.9
	Private	1.5	2.5	14.5	2.8	4.8	2.2	11.8	4.6
	Public	8.9	14.5	58.8	12.0	17.5	11.8	44.2	16.9
	University	1.2	2.8	12.0	0.8	4.9	4.6	16.9	1.5
**A-Netherlands**	Non-profit	0.2	0.8	3.5	0.6	3.8	2.9	9.4	2.4
	Private	0.8	1.7	6.9	0.8	2.9	3.4	9.3	3.0
	Public	3.5	6.9	60.9	8.8	9.4	9.3	44.9	9.8
	University	0.6	0.8	8.8	0.9	2.4	3.0	9.8	1.8
**A-Norway**	Non-profit	0.1	1.5	5.0	2.3	1.1	1.4	4.1	0.3
	Private	1.6	6.8	15.4	3.4	1.4	5.2	17.2	1.8
	Public	5.0	15.4	43.4	18.0	4.1	17.2	50.4	10.9
	University	2.3	3.4	18.0	1.6	0.3	1.8	10.9	1.5
**All three affiliations**	Non-profit	0.5	1.3	5.8	1.1	1.9	2.5	9.8	2.7
	Private	1.3	3.0	11.5	2.1	2.5	3.2	11.8	3.3
	Public	5.8	11.5	56.1	11.9	9.8	11.8	45.2	12.9
	University	1.1	2.1	11.9	1.0	2.7	3.3	12.9	1.6

Percentage share of articles with two or more funders acknowledged, and two or more authors; figures do not necessarily sum to 100% (cells are non-exclusive); A = affiliated.

Many ‘public-public’ amalgamations included the EU (coded as a supranational, public instrument) plus a national public instrument. Public-private (i.e. companies) and public-university amalgamations were also present. Norway-affiliated articles had the highest prevalence of these; Netherlands-affiliations had the lowest. Amalgamations with no public funding instruments were rare. For Denmark-affiliations these accounted for less than 10 percent of amalgamations; for Netherlands and Norway-affiliations, less than 20 percent.

Patterns of funding amalgamations by instrument origin are presented in Table 7. Across all three affiliations, in both fields, the most prevalent amalgamation was ‘domestic-domestic’ (32.5% for Renewable Energy Research; 34.2% for Food Science). There were amalgamations with no domestic funding acknowledged. This indicates papers funded exclusively by amalgamations of foreign and/or supranational funders. Amalgamations in papers with Netherlands-affiliated authors reported the fewest domestic funders.

**Table 7:** Patterns of funding amalgamations by funding origin, 2009–18.

		Renewable Energy Research			Food Science		
		Domestic	Foreign	Supranational	Domestic	Foreign	Supranational
**A-Denmark**	Domestic	35.8	29.4	21.8	40.3	23.7	17.0
	Foreign	29.4	28.2	15.3	23.7	25.3	15.2
	Supranational	21.8	15.3	2.4	17.0	15.2	1.8
**A-Netherlands**	Domestic	29.8	23.0	21.6	23.9	20.6	14.5
	Foreign	23.0	24.7	24.4	20.6	26.6	19.7
	Supranational	21.6	24.4	4.9	14.5	19.7	2.4
**A-Norway**	Domestic	33.1	29.7	15.0	40.6	23.5	13.5
	Foreign	29.7	25.2	12.3	23.5	21.0	15.2
	Supranational	15.0	12.3	1.1	13.5	15.2	2.2
**All three affiliations**	Domestic	32.5	25.8	19.7	34.2	20.3	13.6
	Foreign	25.8	24.7	17.1	20.3	21.5	14.7
	Supranational	19.7	17.1	3.0	13.6	14.7	1.4

Share of articles, two or more funders acknowledged, and two or more authors. Figures may not tally to 100% due to multiple possible, non-exclusive ‘origin-origin’ and other origin permutations; A = affiliated.

There were also ‘domestic-foreign’ and ‘domestic-supranational’ amalgamations. These are country-crossing co-funded publications, funded by funders operating in different countries or at differing geographic scales. Further research could determine if this was intentional action, e.g. funder-to-funder coordination efforts or international research projects, or due to separate dynamics such as researcher collaborations not attributable to the acknowledged funding.

#### Co-funding networks

Co-funding networks for the two fields present dynamics that stretch beyond national landscapes. They differ from what might be visualised via nationally-supplied research funding statistics or perspectives. They present an overall view of co-use of funding at the level of researchers active in a field, built upon funding co-use by discrete researcher collaborations (amalgamations) and co-use by individual researchers (configurations). At this level, co-used instruments can also be grouped according to the funders providing them, in addition to labelling of instrument type and origin.

**Fig 3:**
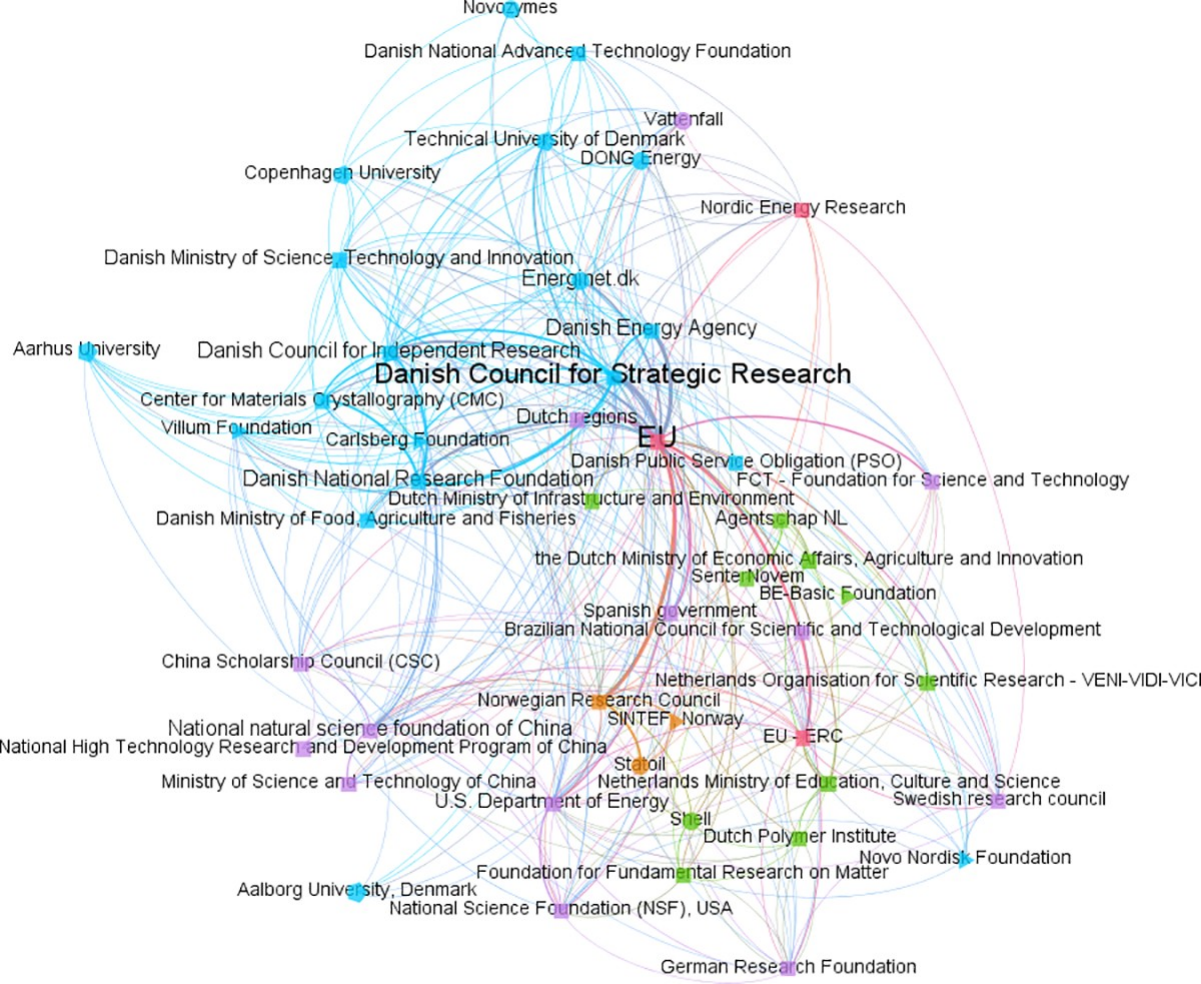
Co-funding network for Renewable Energy Research, all affiliation countries, articles 2009–18. Origin colours: Blue = affiliated-Denmark; green = affiliated-Netherlands; orange = affiliated-Norway; purple = affiliated other countries; red = supranational. Type shapes: Triangle = non-profit; square = public; circle = private; pentagon = university. Node size = number of FAs mentioning that funding instrument; ties = instrument co-occurrences within the same article’s FAs.

**Fig 4:**
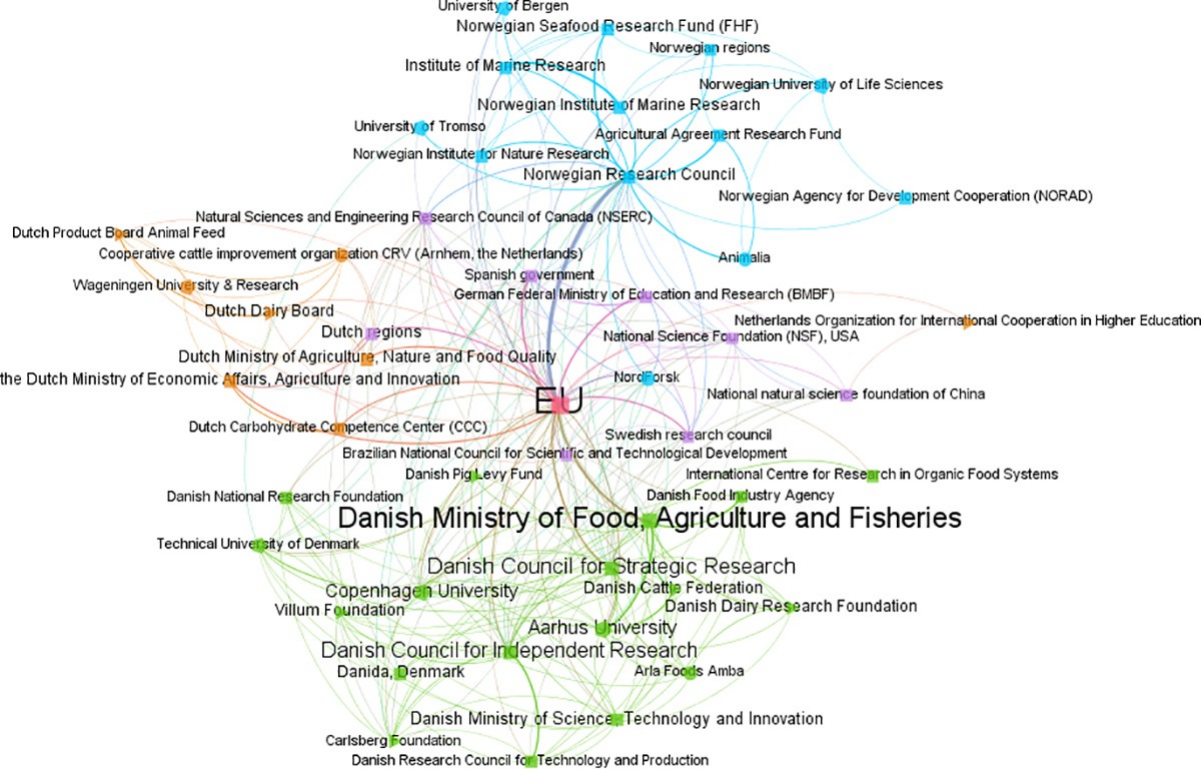
Food Science co-funding network, all affiliation countries. Origin colours: Green = affiliated-Denmark; orange = affiliated-Netherlands; blue = affiliated-Norway; purple = affiliated other countries; red = supranational. Other labels as per Fig 3.

Fig 3 presents the Renewable Energy Research co-funding network across all author country affiliations; Fig 4 shows the Food Science co-funding network for all affiliations. Both networks have many instances of multiple, rather than simple, ties between funding instrument types (indicated by node shape; with funder names also shown) and origins (shown by node colour).

Diverse associations emerge from these visual representations. For example, for Fig 3, a first noticeable feature is the centrality of the EU within the co-funding field network. Such a focal position is emphasized by its node size, indicating the number of FAs mentioning any EU funding instrument, and by multiple links converging at this particular node. The distribution of the most acknowledged funders, to some extent, is also displayed in the highly interconnected sub-network areas. These are populated by funder names from each of the three specific study countries. Among them, particular funding agencies, such as the Danish Council for Strategic Research, seem to play a significant role. Research funding from other countries (e.g. China, USA, Sweden or Spain) may stem from internationalization patterns in publication co-authorships (this would require further study). Collaborative knowledge production is reflected in the co-use of funding instruments from different types and origins, to generate scientific outputs in a field. Varied and mixed combinations (e.g. public-public/supranational-foreign features) are also evident, similarly in Fig 4. This reveals a fine-grained view of funding at the top level of our approach. Separate methods could in future be used to determine the degree to which the network positions and apparent roles of each funder here are strategic, i.e. a conscious result of some funder actions or deliberate funding instrument designs. The co-funding network could be used to begin such an exploration, e.g. interviews with funders–perhaps also combined with insights from the observed funding amalgamations and configurations.

Similarly, assorted type-based ties are present in both Figs 3 and 4. Specific private companies and non-profit organizations are identifiable among the variety of research funding acknowledged across all publications in both fields. For instance, in Fig 3 we see energy-related organizations, and in Fig 4, food-related ones. Within the Fig 3 network, as an example, numerous university type instruments (internal funding) are linked together, as well as simultaneously to multiple varieties of public, private and non-profit instruments (e.g. Technical University of Denmark instruments linked to those of Aarhus University, the Novo Nordisk and Carlsberg foundations, Danish or Norwegian public agencies). In other words, researchers here are engaged in heterogeneous funding co-use (similarly to their configurations and amalgamations, but now at a higher level).

The full dataset can be filtered to present other related funding dynamics to study, i.e. to show field co-funding networks filtered by author country affiliation. These country-filtered co-funding networks still present highly heterogeneous funding dynamics, due to how they have been built from configurations and amalgamations, rather than top down. For our cases, traditional public funding sources, like the EU, remain prevalent once the networks are filtered by affiliation countries, but are clearly operating as parts of large networks of interlinked funding instruments, and not in isolation.

Specific features are also present when the networks are filtered by affiliations. For instance, when filtered for Norway-affiliated researchers, the Norwegian Research Council is shown to have a ‘hub’ function. This may be expected for a centralised public funder, but here can be visualized as occurring from researchers actually co-using their funding, in some way, to collaborate to produce research outputs (irrespective of whether such activity was strategically intended by funders). However, even when filtered this way, ties between more traditional national public funders and non-public, foreign or supranational funders are also present. This emphasizes the interconnected nature of the funding. Field-specific funders stand out for certain cases, e.g. oil companies in the Norway-affiliated Renewable Energy Research case. Additionally, for all six cases, filtered co-funding networks show smaller/niche funders that might be overlooked in a top-down perspective.

This filtering approach underlines that the bottom-up approach does not have to discard entirely the notion of countries. National funding remains present and can still be studied. National funders appear important across all cases, even with the noted emergence generally of multi-level, multi-actor ‘global science’ [c.f. 11]. The bottom-up approach can also provide more nuance here, and show that traditional funders nevertheless do however operate in heterogeneous, variously interlinked ways with other funders’ instruments, due to multiple levels of co-use by researchers.

Overall, the bottom-up approach enables study of potentially useful insights into seemingly highly complex funding situations. Future research using the same approach could go further, e.g. to explore field-nexus contexts, e.g. a two field, energy-and-food co-funding network. This would make sense primarily in instances where research topics overlap between fields. For such cases, the approach could reveal unexpected linkages. Similarly, the co-funding networks could be filtered according to author affiliation(s). This would enable study of ranked prevalence and variety of named funders/funding instruments, relevant to country affiliation related aspects.

## Discussion and conclusion

Overall, the findings presented above support the key assumption in this paper: taking a researcher-led perspective to get to the ‘bottom’ of research funding can reveal important, varied funding co-use dynamics. There are also several other implications from testing this bottom-up approach.

First, for the test fields of Renewable Energy Research and Food Science, the configuration, amalgamation and co-funding network levels present funding co-use dynamics one would expect from globalisation of science or ‘global science’ [11], but add more nuance and granularity. The bottom-up perspective, as tested here, verifies recent assertions that knowledge production is increasingly no longer (if indeed it ever was) the ‘product’ of any particular, single country or national science system. This assertion holds across co-use of funding by individual researchers (configurations), collaborating researchers (amalgamations), and fields of collaborating researchers (co-funding networks). It implies not only individual researchers and research organizations navigate an increasingly complex globalised science funding system, but also that funders need to understand and adapt to this complicated operational context, featuring highly interlinked, multi-level, country border-crossing and seemingly interdependent funding co-uses [see 12]. Further research using the bottom-up approach for more fields, and for other collaborative research activities not just limited to research in the form of publications (and funding registering via FAs), could generate more general insights about funding dynamics, throughout more of the global science system.

Second, the findings support the assertion that a bottom-up, researcher-led perspective on research funding is valid and necessary. The approach provides useful analytical units that aggregate funding at meaningful levels (researchers, collaborators, fields). These add nuance and help to understand more about the nature of contemporary, interlinked research funding realities in various fields and contexts. They have been tested here using FAs and focusing on publications, but need not be limited to this. At the same time, the framing and test may bring new opportunities for further exploration of the possible complementarities that bottom-up findings could contribute to top-down/system-led studies.

Third, the test presented a high heterogeneity of funding co-use. Domestic, foreign and supranational funders from public, private, university and non-profit sectors were configured, amalgamated and networked in various ways. The implication for understanding funding is that researchers (either individual, collaborating or within a field) can co-use assorted funding instruments to do research. Certain national and sectoral specificities remain present, but geographic boundary-spanning dynamics seem now inherent. This implies future studies of research funding not only need to assign continued importance to national funding but also address funding co-use that can be present right from the bottom up, for individual researchers and beyond. In other words, this bottom-up approach is not simply intended to be a descriptive analysis of funding/funders, similar to what is already possible with scientometrics approaches. It is an attempted re-framing, aiming to stress that there are additional, important levels at which of co-use funding by researchers needs to be considered, because of increasing complexity in contemporary funding dynamics.

Fourth, the bottom-up approach could be used to generate intelligence for funders, policymakers, university research managers and so on. The approach is grounded in funding as it is actually co-used. This is independent of whether such use has been strategically coordinated, intended or expected by various stakeholders [c.f. 35, 47, 48, 65]. This perspective could potentially enable funders to look more inclusively and pragmatically at their funding portfolios, to assess and adjust their bi- or multi-lateral coordination efforts, and perhaps to understand better their potential co-influence throughout the science system [c.f. 39, 44, 52].

At the same time, two limitations need to be highlighted. First, the selected fields to test the approach are highly applied and interdisciplinary. These cases may have limited generalisability. Specifically, they might have idiosyncratic knowledge production and collaborations that are not relevant elsewhere (e.g. in mono-disciplinary fields). Their similarity was useful in this paper to supply funding variety to test the multi-level funding co-use approach. For wider applicability, the approach should be tested not only with more but also with different fields. Similarly, the configuration, amalgamation and co-funding network patterns presented in this paper are obviously specific to the two illustrative fields and for Denmark, Netherlands or Norway affiliated researchers only (i.e. these are researchers in small but open economies that already have strong international ties). Therefore, these cases likely affect how complex and country boundary-spanning co-use appears here.

A second limitation concerns the selected collaborative research activity of research outputs/publications, and use of FAs as the data source for the test and operationalization of our approach. Publication FAs do provide relevant data. However, they do not afford fine details to unpack funding dynamics. Improving guidelines around author use of FAs could potentially contribute to this end, e.g. journals could mandate clear, robust author-funding attribution. The bottom-up approach could then better study the co-use dynamics of funding amalgamations, using this additional funding-author attribution data. Nevertheless, it would remain challenging to attribute particular discrete research content or insights within any given article to particular funding, even with direct author-funding attribution. Further research would still be needed to provide understanding of whether collaborating authors used their own funding in isolation (amalgamations juxtaposing independent funding instruments) or collectively (amalgamations fusing interdependent instruments).

The three levels of the bottom-up analysis could also be differently explored. Future research could use additional methods to study, for instance, what funding configurations exist in particular fields and contexts–or how different types and origins of funding instruments are co-used within configurations. As we have stated, this aggregation level of funding co-use has been largely overlooked in previous studies. And yet, as we have illustrated, even for a small sub-sample, configurations can exist as a distinct level of funding co-use. They can also be varied, warranting further research attention. This clearly requires use of a different method than tracing funding via FAs, and a sole focus on papers. This can result in insufficient numbers of configurations to study (i.e. single-authored papers), particularly in fields where publication is primarily collaborative (multi-authored). Additionally, for funding amalgamations, their composition and how instruments are co-used are other potential research interests. In particular, the inability of a FA-based empirical exploration to distinguish between fused or juxtaposed amalgamations, invites further research using additional methods. Likewise, variation in patterns of co-funding networks in different field types, in field-spanning contexts, or in emerging research areas located in-between existing fields, could also be investigated. For instance, here it could be explored whether network patterns correlate with deliberate funder strategies to achieve synergy through funding co-use within a given field. There could also be separate but related questions to explore, including whether and how researchers enact agency to pursue strategies to (co-)shape the availability and characteristics of funding in a field, which researchers later assemble into funding configurations, amalgamations, and co-funding networks. This could explore if researchers attempt to influence funders to affect funding provision that they need to co-use to do their research in particular ways, in their field [c.f. 5, 14].

Expanding the approach could include additional methods like interviews, case studies, bibliometrics, altmetrics, surveys and/or network analysis. The approach presented in this paper clearly remains instrumental. It has been an initial means to these kinds of analytical ends, not a final goal in itself. Next steps could add further funding variables or characteristics to move beyond the descriptive, technical classifications of type and origin. Further research could attempt to determine what is distinctive about funding instruments apart from these labels, in a given context, which was beyond the scope of this paper. Examples could be features of autonomy and flexibility of instruments, their duration, amount, or focus on impact/users–and whether these matter more in some fields than others. Additionally, whether and how researchers respond to such characteristics of funding provision through configuration and amalgamation-related behaviours could also be explored.

For this current paper, the bottom-up, co-use of funding by researchers approach has provided both a ‘proof-of-concept’ of the perspective, and a foundation for such future studies. For the approach to provide more insights, and for it to be generalisable across the science system, it should ideally comprise part of a broader, multi-faceted framework and mixed methods toolkit. This could then facilitate detailed study and better understanding of a wide range of the effects of contemporary research funding dynamics upon researchers and their research.

## Supporting information

S1 File Dataset core/expansion further details. List of core keywords, relevant journals, and article-level clusters for the dataset of Renewable Energy Research and Food Science publications.
(DOCX)

S2 File Dataset. Listing anonymised unique article identifiers, author affiliations, numbers of authors, fields, numbers of funding acknowledgements, and origin and type data used in this paper.
(XLSX)
