# Supplementary material for: Getting to the bottom of research funding: Acknowledging the complexity of funding dynamics
Source: PLoS One. 2021 May 12;16(5):e0251488. doi: 10.1371/journal.pone.0251488 (PMC8115833; doi:10.1371/journal.pone.0251488)
Supplement: S1 File — List of core keywords, relevant journals, and article-level clusters for the dataset of Renewable Energy Research and Food Science publications. (DOCX) [file pone.0251488.s001.docx]

**S1 File:**

**Core keywords, journals, and article-level clusters for ‘Food Science’**

**Keywords**

green farm*, sustainable farm*, renewable farm*, renewable food*, green food*, sustainable food*, green agr*, sustainable agr*, renewable agr*, food*, animal*, fish*, crop*, dairy*

**Journals**

Acta agriculturae scandinavica section a-animal science, Acta alimentaria, African journal of agricultural research, Agrarforschung, Agrarforschung schweiz, Agrekon, Agribusiness, Agricultural and food science, Agricultural economics, Agricultural economics-zemedelska ekonomika, Agricultural history, Agricultural sciences in china, Agricultural systems, Agriculture and human values, Agriculture ecosystems & environment, Agrociencia, Agroecology and sustainable food systems, Ama-agricultural mechanization in asia africa and latin america, American journal of agricultural economics, American journal of enology and viticulture, Analytical methods, Animal, Animal biotechnology, Animal feed science and technology, Animal frontiers, Animal genetics, Animal nutrition and feed technology, Animal production science, Animal reproduction, Animal reproduction science, Animal science journal, Animal science papers and reports, Animals, Annals of animal science, Annals of applied biology, Annual review of animal biosciences, Annual review of food science and technology, Annual review of resource economics, Antioxidants, Applied animal behaviour science, Applied biological chemistry, Applied economic perspectives and policy, Applied engineering in agriculture, Aquacultural engineering, Archiv fur geflugelkunde, Archiv fur tierzucht-archives of animal breeding, Archives animal breeding, Archives of animal nutrition, Asian-australasian journal of animal sciences, Australian journal of agricultural and resource economics, Australian journal of dairy technology, Australian journal of grape and wine research, Avian biology research, Berichte uber landwirtschaft, Biomass & bioenergy, Bioresource technology, Bioscience biotechnology and biochemistry, Bioscience journal, Biosystems engineering, Biotechnology progress, Boletim do centro de pesquisa de processamento de alimentos, Bragantia, Brazilian journal of poultry science, British food journal, British poultry science, Buffalo bulletin, Bulgarian journal of agricultural science, Cahiers agricultures, California agriculture, Canadian journal of agricultural economics-revue canadienne d agroeconomie, Canadian journal of animal science, Cereal chemistry, Cereal foods world, Chemical senses, Chemosensory perception, Chilean journal of agricultural research, China agricultural economic review, Ciencia e agrotecnologia, Ciencia e investigacion agraria, Ciencia e tecnica vitivinicola, Comprehensive reviews in food science and food safety, Computers and electronics in agriculture, Critical reviews in food science and nutrition, Crop & pasture science, Cuadernos de desarrollo rural, Cuban journal of agricultural science, Current opinion in food science, Custos e agronegocio on line, Cyta-journal of food, Czech journal of animal science, Czech journal of food sciences, Dairy science & technology, Deutsche lebensmittel-rundschau, Domestic animal endocrinology, Elelmiszervizsgalati kozlemenyek, Emirates journal of food and agriculture, Engenharia agricola, European food research and technology, European journal of lipid science and technology, European poultry science, European review of agricultural economics, Flavour and fragrance journal, Fleischwirtschaft, Food & function, Food & nutrition research, Food additives & contaminants part b-surveillance, Food additives and contaminants part a-chemistry analysis control exposure & risk assessment, Food analytical methods, Food and agricultural immunology, Food and bioprocess technology, Food and bioproducts processing, Food and chemical toxicology, Food and drug law journal, Food and energy security, Food and nutrition bulletin, Food australia, Food biophysics, Food bioscience, Food biotechnology, Food chemistry, Food control, Food engineering reviews, Food hydrocolloids, Food hygiene and safety science, Food microbiology, Food packaging and shelf life, Food policy, Food quality and preference, Food research international, Food reviews international, Food science & nutrition, Food science and biotechnology, Food science and human wellness, Food science and technology, Food science and technology international, Food science and technology research, Food security, Food structure-netherlands, Food technology, Food technology and biotechnology, Foodborne pathogens and disease, Foods, Fourrages, Genetics selection evolution, German journal of agricultural economics, Global food security-agriculture policy economics and environment, Grasas y aceites, Grassland science, Icelandic agricultural sciences, Indian journal of agricultural sciences, Indian journal of animal research, Indian journal of animal sciences, Industrial crops and products, Innovative food science & emerging technologies, Inra productions animals, International dairy journal, International food and agribusiness management review, International food research journal, International journal of agricultural and biological engineering, International journal of agricultural sustainability, International journal of agriculture and biology, International journal of dairy technology, International journal of food engineering, International journal of food microbiology, International journal of food properties, International journal of food science and technology, International journal of food sciences and nutrition, International sugar journal, Irish journal of agricultural and food research, Italian journal of animal science, Italian journal of food science, Itea-informacion tecnica economica agraria, Jarq-japan agricultural research quarterly, Journal fur verbraucherschutz und lebensmittelsicherheit-journal of consumer protection and food safety, Journal of agricultural & environmental ethics, Journal of agricultural and food chemistry, Journal of agricultural and resource economics, Journal of agricultural economics, Journal of agricultural meteorology, Journal of agricultural science, Journal of agricultural science and technology, Journal of agricultural sciences-tarim bilimleri dergisi, Journal of agriculture and rural development in the tropics and subtropics, Journal of agriculture of the university of puerto rico, Journal of animal and feed sciences, Journal of animal and plant sciences, Journal of animal breeding and genetics, Journal of animal physiology and animal nutrition, Journal of animal science, Journal of animal science and biotechnology, Journal of aoac international, Journal of applied animal research, Journal of applied poultry research, Journal of aquatic food product technology, Journal of bioscience and bioengineering, Journal of cereal science, Journal of consumer protection and food safety, Journal of dairy research, Journal of dairy science, Journal of essential oil research, Journal of food agriculture & environment, Journal of food and drug analysis, Journal of food and nutrition research, Journal of food biochemistry, Journal of food composition and analysis, Journal of food engineering, Journal of food lipids, Journal of food measurement and characterization, Journal of food process engineering, Journal of food processing and preservation, Journal of food protection, Journal of food quality, Journal of food safety, Journal of food safety and food quality-archiv fur lebensmittelhygiene, Journal of food science, Journal of food science and technology-mysore, Journal of functional foods, Journal of integrative agriculture, Journal of irrigation and drainage engineering, Journal of land use science, Journal of medicinal food, Journal of muscle foods, Journal of oil palm research, Journal of oleo science, Journal of plant diseases and protection, Journal of poultry science, Journal of reproduction and development, Journal of sensory studies, Journal of sustainable agriculture, Journal of texture studies, Journal of the american oil chemists society, Journal of the american society of brewing chemists, Journal of the faculty of agriculture kyushu university, Journal of the institute of brewing, Journal of the japanese society for food science and technology-nippon shokuhin kagaku kogaku kaishi, Journal of the korean society for applied biological chemistry, Journal of the science of food and agriculture, Journal of wine economics, Korean journal for food science of animal resources, Landbauforschung, Large animal review, Listy cukrovarnicke a reparske, Livestock science, Lwt-food science and technology, Meat science, Milchwissenschaft-milk science international, Mitteilungen klosterneuburg, Mljekarstvo, Molecular nutrition & food research, Natural product communications, New medit, New zealand journal of agricultural research, Njas-wageningen journal of life sciences, Oeno one, Outlook on agriculture, Packaging technology and science, Paddy and water environment, Pakistan journal of agricultural sciences, Pesquisa agropecuaria brasileira, Philippine agricultural scientist, Plant foods for human nutrition, Plant pathology journal, Polish journal of food and nutrition sciences, Postharvest biology and technology, Poultry science, Precision agriculture, Quality assurance and safety of crops & foods, Renewable agriculture and food systems, Reproduction in domestic animals, Review of agricultural economics, Revista brasileira de engenharia agricola e ambiental, Revista brasileira de zootecnia-brazilian journal of animal science, Revista ciencia agronomica, Revista colombiana de ciencias pecuarias, Revista de la facultad de ciencias agrarias, Revista mexicana de ciencias pecuarias, Revista mvz cordoba, Revue suisse d agriculture, Rivista italiana delle sostanze grasse, Sciences des aliments, Scientia agricola, Semina-ciencias agrarias, Small ruminant research, South african journal of animal science, South african journal of enology and viticulture, Spanish journal of agricultural research, Starch-starke, Sugar industry-zuckerindustrie, Tarim bilimleri dergisi-journal of agricultural sciences, Tecnica pecuaria en mexico, Transactions of the asabe, Trends in food science & technology, Tropical agriculture, Tropical animal health and production, Tropical grasslands-forrajes tropicales, World mycotoxin journal, World rabbit science, Worlds poultry science journal, Zemdirbyste-agriculture, Zuchtungskunde, Zywnosc-nauka technologia jakosc

**Article-level clusters**

| ***Cluster #*** | ***Top terms in clusters*** |
| --- | --- |
| 521 | anthocyanins; cyanidin; euterpe oleracea mart; blueberry; strawberry |
| 540 | marine spatial planning; governance; coastal management; marine reserf; small scale fishery |
| 832 | collembola; oligochaeta; acari; megascolecidae; lumbricidae |
| 975 | hemocyte; crassostrea virginica; pacific oyster; ruditapes philippinarum; eastern oyster |
| 1010 | glycemic load; risk; diabetes; rat; satiety |
| 1594 | inactivation; high hydrostatic pressure; high pressure processing; electric field; escherichia coli |
| 1632 | florfenicol; oxytetracycline; antibiotic residue; quinolone; chloramphenicol residue |
| 1925 | food; gmo; organism; biotechnology; gene flow |
| 1960 | agriculture; diet; meat; farm; organic farming |
| 2202 | intercropping; weed suppression; fertility level; brassica juncea; rainfed condition |
| 2312 | rice intensification; chlorophyll meter; alternate wetting; sri; ammonia volatilization |
| 2390 | deep fat frying; oil uptake; frying; potato chip; french fry |
| 2812 | potato tuber; solanum tuberosum l; glycoalkaloid; tuber; antioxidant activity |
| 2993 | beta galactosidase; lactose hydrolysis; human milk oligosaccharide; sialic acid; lactose |
| 3328 | acrylamide; glycidamide; potato chip; french fry; asparagine |
| 3370 | dairy product; dairy consumption; calcium; lactose intolerance; metabolic syndrome |
| 3544 | ice cream; perception; aroma release; saliva; headspace gas chromatography |
| 4021 | edible insect; tenebrio molitor; black soldier fly; growth performance; hermetia |
| 3578 | lepeophtheirus salmonis; sea lice; siphonostomatoida; caligidae; salmon louse |
| 3273 | food sovereignty; land grab; agroecology; large scale land acquisition; dispossession |
| 2771 | cassava; manihot esculenta crantz; tarhana; sourdough; euphorbiaceae |
| 2590 | odor; livestock building; broiler house; ammonia emission; hydrogen sulfide |
| 2272 | cocoa butter; enzymatic interesterification; diacylglycerol; palm stearin; lipase |
| 2111 | bangladesh; biofloc; periphyton; water quality; environmental impact |
| 2040 | machine vision; computer vision; image processing; vacuum cooling; image analysis |
| 1675 | boar taint; sow; piglet; androstenone; entire male pig |
| 1674 | sulforaphane; glucosinolate; broccoli; indole; carbinol |
| 1620 | digital dermatitis; footrot; lameness; dichelobacter nodosus; animal welfare |
| 1474 | campylobacter coli; arcobacter butzleri |
| 1458 | angiotensin; enzyme inhibitory peptide; ace; bioactive peptide; hypertensive rat |
| 1451 | white clover; seed yield; trifolium; structural characteristic; n alkane |
| 1375 | climate change; climate variability; crop model; vulnerability; aquacrop |
| 1129 | bovine mastitis; staphylococcus aureus; intramammary infection; somatic cell count; coagulase negative staphylococci |
| 983 | conservation agriculture; maize; technology adoption; western kenya; smallholder farm |
| 969 | genistein; isoflavone; daidzein; soy; phytoestrogen |
| 952 | nitrous oxide emission; denitrification; methanotroph; n2o emission; n2o |
| 902 | pectin; feruloyl esterase; pectic polysaccharide; xyloglucan; plant cell wall |
| 808 | high molecular weight glutenin subunit; dough; bread dough; dough rheology; wheat flour dough |
| 802 | volatile compound; wine; ethyl carbamate; oenococcus oeni; malolactic fermentation |
| 778 | advanced glycation end product; glyoxalase; methylglyoxal; glycation; ages |
| 765 | growth performance; aeromonas hydrophila; disease resistance; flavobacterium psychrophilum; aeromonas |
| 755 | varroa destructor; megachilidae; halictidae; apoidea; nosema ceranae |
| 702 | pig; phytase; acid digestibility; microbial phytase; soluble |
| 688 | naringenin; nobiletin; quercetin; fisetin; hesperetin |
| 675 | tenderness; sex; carcass characteristic; carcass; breed |
| 647 | growth trait; random regression model; genetic evaluation; pedigree analysis; genetic parameter estimate |
| 636 | stanniocalcin; hypoxia; osmoregulation; clove oil; eugenol |
| 535 | pesticide residue; dissipation; uncertainty; measurement uncertainty; grape |
| 487 | incubation; feather pecking; hatchability; furnished cage; ascite |
| 440 | soybean meal; fish meal; fatty acid composition; practical diet; digestibility |
| 426 | glyphosate resistance; amaranthus palmeri; selectivity; translocation; conyza canadensis |
| 416 | microbial transglutaminase; surimi; fillet; gelation; rainbow trout |
| 398 | air water interface; complex coacervation; water emulsion; cultivar; soybean |
| 380 | rice starch; amylopectin; granule; heat moisture treatment; adp glucose pyrophosphorylase |
| 359 | lactococcus lactis; streptococcus; cheddar cheese; cheese; casein micelle |
| 342 | progesterone; corpus luteum; mare; estrous cycle; luteolysis |
| 278 | solvent extract; total antioxidant capacity; total phenolic content; dpph; antioxidant potential |
| 271 | methylcyclopropene; polyphenol oxidase; shelf life; ethylene; mcp |
| 267 | rhizobacteria; trichoderma spp; growth promotion; bacillus subtili; plant growth |
| 174 | length weight relationship; sardinops sagax; atlantic bluefin tuna; anchovy; thunnus thynnus |
| 145 | rice; oryza sativa l; cold tolerance; qtl detection; quantitative trait loci |
| 120 | ochratoxin; aflatoxin; deoxynivalenol; zearalenone; mycotoxin |
| 45 | aerobic stability; methane production; corn silage; methane emission; microbial protein synthesis |

**Core keywords, journals, and article-level clusters for ‘Renewable Energy Research’**

**Keywords**

renewable energ*, sustainable energ*, *power conver*, alternative energ*, electricity generat*, electricity system*, renewable*, sustainab*, bioenergy, alternative fuel*, biodiesel*, biofuel*, biogas*, biomass combustion, biomass gasification, biomass power, biomass production, biomass pyrolysis, biochar*, bioethanol, biomass energ*, biomass fuel*, biomass to energy, energy from biomass, palm oil, soybean oil, thermal water, hydro turbine*, hydro energ*, tidal energ*, tidal power, water power, tidal stream, wave energ*, wave power, photovoltaic generat*, photovoltaic module*, photovoltaic system*, solar air, solar concentrator*, solar energ*, solar thermal, photovoltaic*, solar thermal collector*, wind farm*, wind park*, wind turbine*, wind energ*, wind generat*, wind power, wind turbine generat*, **Walney Extension, London Array, Gemini Wind Farm, Anholt, BARD Offshore 1, Gwynt y Môr, , ethanol biofuel*, ethanol, cellulosic ethanol, algae fuel*, Jatropha, PV, PV system*, PV power*, crystalline silicon, zero-emission fuel*, fuel-cell*, Hydrogen fuel*, steam reforming, steam-methane reforming, Green energ***

Note: **Bold** keywords are from: Rizzi F, van Eck NJ, Frey M. The production of scientific knowledge on renewable energies: Worldwide trends, dynamics and challenges and implications for management. Renewable Energy. 2014;62:657-71.

**Journals**

Algal research-biomass biofuels and bioproducts, Alternative sources of energy, Bioenergy research, Biofuels bioproducts & biorefining-biofpr, Biofuels-uk, Biomass & bioenergy, Biomass conversion and biorefinery, Biomass, Bioresource technology, Biotechnology for biofuels, Energy & environment, Energy & environmental science, Energy for sustainable development, Energy sustainability and society, Environmental progress & sustainable energy, Fuel cells, Geothermal energy, Geothermics, Global change biology bioenergy, Ieee journal of photovoltaics, Ieee transactions on sustainable energy, Iet renewable power generation, International journal of exergy, International journal of green energy, International journal of hydrogen energy, International journal of photoenergy, Journal of fuel cell science and technology, Journal of modern power systems and clean energy, Journal of photonics for energy, Journal of renewable and sustainable energy, Journal of solar energy engineering-transactions of the asme, Progress in photovoltaics, Renewable & sustainable energy reviews, Renewable energy, Solar age, Solar cells, Solar energy materials and solar cells, Solar energy materials, Solar energy, Sustainable energy & fuels, Sustainable energy grids & networks, Sustainable energy technologies and assessments, Wiley interdisciplinary reviews-energy and environment, Wind energy

**Article-level clusters**

| ***Cluster #*** | ***Top terms in cluster*** |
| --- | --- |
| 58 | polymer solar cells; open circuit voltage; bulk heterojunction solar cell; photovoltaic performance; power conversion efficiency |
| 1889 | wind turbine; wind farm; vertical axis wind turbine; large eddy simulation; complex terrain |
| 186 | hydrogen production; anaerobic co digestion; food waste; dark fermentation; hydrogen |
| 908 | switchgrass; biofuel; ethanol; miscanthus; energy crop |
| 1275 | wind energy; wind; offshore wind farm; feed; energy return |
| 2714 | sustainability transition; public procurement; multi level perspective; transition management; grassroots innovation |
| 60 | beta glucosidase; bioethanol production; cellulase; endoglucanase; enzymatic hydrolysis |
| 347 | corporate social responsibility; iso; csr; environmental performance; stakeholder |
| 668 | light trapping; crystalline silicon solar cell; screen; broadband; absorption enhancement |
| 922 | electric vehicle; vehicle; plug; smart home; demand side management |
| 115 | zirconia; cerium; yttria; intermediate temperature solid oxide fuel cell; lasr |
| 706 | unit commitment problem; market power; transmission expansion planning; economic dispatch problem; valve point effect |
| 599 | microalgae; chlorella vulgaris; photobioreactor; nutrient removal; light intensity |
| 674 | islanded microgrid; islanding detection; inverter; reconfiguration; distribution network reconfiguration |
| 1930 | dfig; induction generator; wind turbine; variable speed wind turbine; wind farm |
| 603 | bio oil; hydrothermal liquefaction; hydrodeoxygenation; bio oil production; torrefaction |
| 615 | climate sensitivity; international environmental agreement; emissions trading; geoengineering; carbon tax |
| 88 | ethanol oxidation; pt c catalyst; pt nanoparticle |
| 1040 | microbial fuel cell; performance; electricity generation; bioelectrochemical system; shewanella oneidensis mr |
| 1640 | biochar; effect; soil; hydrothermal carbonization; characterization |
| 888 | bipolar plate; gas diffusion layer; pem fuel cell system; phase flow; micro direct methanol fuel cell |
| 552 | perovskite solar cell; stability; perovskite; performance; efficiency |
| 1854 | wave energy converter; array; layer fluid; breakwater; water wafe |
| 331 | esterification; combustion; diesel engine; emission characteristic; diesel |
| 944 | ethanol steam; syngas production; methane decomposition; catalytic partial oxidation; dry reforming |
| 546 | proton conductivity; arylene ether sulfone; sulfonated poly; vinyl alcohol; composite membrane |
| 1709 | turkey; hydrogen production; hydrogen; algeria; hybrid renewable energy system |
| 2302 | emergy; emergy analysis; emergy evaluation; degrowth; ecological footprint |
| 2516 | molten carbonate fuel cell; mcfc; solid oxide fuel cell system; heat; hybrid system |
| 752 | environmental kuznets curve; financial development; decomposition analysis; economic growth; energy consumption |
| 421 | coal pyrolysis; underground coal gasification; biomass gasification; coal char; nox emission |
| 65 | porous silicon; silicon nanocrystal; microcrystalline silicon; h thin film; silicon quantum dot |
| 1546 | solar air heater; pv t; absorber plate; photovoltaic thermal system; compound parabolic concentrator |
| 898 | beta o; oxygen delignification; organosolv lignin; kraft lignin; mechanical pulp |
| 2561 | indoor air pollution; rural electrification; solar home system; exposure; child |
| 1722 | high temperature corrosion; corrosion; wood ash; ash deposition; recovery boiler |
| 1210 | organic food; local food; rurality; alternative food network; farmers market |
| 1784 | photovoltaic module; solar cell; partial shading condition; pv module; degradation |
| 585 | electrochemical property; hydrogen storage alloy; alloy; ni mh battery; microstructure |
| 439 | in ga; cu2znsn; sse; cu2znsns4 thin film; cuinse2 |
| 170 | turbulent premixed flame; deflagration; detonation; detonation transition; mild combustion |
| 2310 | butanediol; clostridium acetobutylicum; butanol production; klebsiella pneumoniae; clostridium beijerinckii |
| 125 | dye sensitized solar cell; dssc ; |
| 2491 | proton conductor; hydrogen permeation; bazro3; barium zirconate; proton |
| 1409 | oil spill detection; wind wafe; langmuir circulation; significant wave height; swell |
| 1262 | ammonia borane; hydrogen generation; hydrolysis; sodium borohydride; bh4 |
| 397 | photoelectrochemical water oxidation; solar water splitting; bivo4; hematite; hematite photoanode |
| 82 | photosystem; reaction center; rhodobacter sphaeroide; electron transfer; light harvesting complex |
| 2145 | ground source heat pump system; borehole heat exchanger; soil; ground; groundwater flow |
| 168 | pile; liquefaction; offshore wind turbine; cpt; geogrid |
| 573 | water oxidation; efficient hydrogen evolution; overall water splitting; oxygen evolution reaction; electrocatalytic water oxidation |
| 4204 | luminescent solar concentrator; luminescent; dye; quantum dot; layer |
| 2366 | electrodialysis; anion exchange membrane; bipolar membrane; diffusion dialysis; electrochemical characterization |
| 2232 | global solar radiation; estimation; solar irradiance; satellite; active radiation |
| 205 | fischer tropsch synthesis; co2 methanation; methanol synthesis; cinnamaldehyde; cu zno al2o3 catalyst |
| 1755 | co2 capture; co2 absorption; monoethanolamine; piperazine; co2 |
| 952 | nitrous oxide emission; denitrification; methanotroph; n2o emission; n2o |
| 72 | hzsm; sapo; methanol; zeolite membrane; zeolite catalyst |
| 1378 | scheduling; heat exchanger network; uncertainty; global optimization; planning |
| 2173 | electrochemical reduction; carbon dioxide; co2; co2 reduction; formate |
| 1168 | phase change material; pcm; thermal energy storage; paraffin; latent heat |
| 1426 | efficient oxygen reduction; fe n c catalyst; zn air battery; efficient metal free electrocatalyst; iron phthalocyanine |
| 14 | zno nanorod array; zno film; zno nanowire; zinc oxide thin film; zinc oxide nanorod |
| 3640 | wind farm; vulture; wind turbine; diclofenac; andean condor |
| 3740 | intermediate band solar cell; hot carrier solar cell; quantum dot solar cell; solar cell; impurity photovoltaic effect |
| 396 | chlorophyll content; photochemical reflectance index; desertification; leaf area index; ndvi |
| 1340 | hydrogen production; hydrogenase; methyl coenzyme m reductase; syngas; |
| 2201 | methanol; ethylene glycol; methanol poisoning; activated charcoal; dog |
| 455 | co2 capture; co2 adsorption; carbon dioxide; methane; ch4 |
| 1130 | centrifugal compressor; film; tip clearance; centrifugal fan; tip leakage flow |
| 1974 | hydrogen embrittlement; hydrogen diffusion; hydrogen; high strength steel; hydrogen trapping |
| 2440 | hydrogen production; parabolic trough solar collector; co2; methane; coal |
| 1502 | lead acid battery; charge estimation; electric vehicle; valve; state |
| 249 | gas sensing property; sno2 thin film; sno2 nanostructure; acetone; |
| 2300 | triboelectric nanogenerator; contact electrification; piezo phototronic effect; particle; bed |
| 1767 | organic electrochemical transistor; electrochromic polymer; thermoelectric property; pedotpss film; pedot |
| 2258 | hydrogen storage; molecular hydrogen; storage |
| 1483 | cdte thin film; cds cdte solar cell; cds film |
